# The Isolated and Combined Effects of Folic Acid and Synthetic Bioactive Compounds against Aβ_(25-35)_-Induced Toxicity in Human Microglial Cells

**DOI:** 10.3390/molecules15031632

**Published:** 2010-03-11

**Authors:** Yih-Fong Liew, Chao-Tzu Huang, Shang-Shing P. Chou, Yuh-Chi Kuo, Shiu-Huey Chou, Jyh-Yih Leu, Woan-Fang Tzeng, Su-Jane Wang, Ming-Chi Tang, Rwei-Fen Syu Huang

**Affiliations:** 1 Department of Nutritional Science, Fu-Jen University, 510 Chung-Cheng Rd., Hsinchuang, Taipei County 242, Taiwan; E-Mails: 070647@mail.fju.edu.tw (Y.-F.L.); Psop13@hotmail.com (C.-T.H.); mmiittyy0606@hotmail.com (M.-C.T.); 2 Department of Chemistry, Fu-Jen University, 510 Chung-Cheng Rd., Hsinchuang, Taipei County 242, Taiwan; E-Mail: chem1004@mail.fju.edu.tw (S.S.P.C.); 3 Department of Life Science, Fu-Jen University, 510 Chung-Cheng Rd., Hsinchuang, Taipei County 242, Taiwan; E-Mails: 021553@mail.fju.edu.tw (Y.-C.K); 047751@mail.fju.edu.tw (S.-H.C); 049432@mail.fju.edu.tw (J.-Y.L.); 012098@mail.fju.edu.tw (W.-F.T.); 4 School of Medicine, Fu-Jen University, 510 Chung-Cheng Rd., Hsinchuang, Taipei County 242,Taiwan; E-Mail: med0003@mail.fju.edu.tw (S.-J.W.)

**Keywords:** folate, Aβ_(25-35)_ peptide, microglial cells, newly synthesized compounds, superoxide production, nitric oxide

## Abstract

Folic acid plays an important role in neuronal development. A series of newly synthesized bioactive compounds (NSCs) was reported to exhibit immunoactive and neuroprotective functions. The isolated and combined effects of folic acid and NSCs against β-amyloid (Aβ)-induced cytotoxicity are poorly understood. These effects were tested using human microglia cells (C13NJ) subjected to Aβ_(25-35)_ challenge. According to an MTT assay, treatment of C13NJ cells with Aβ_(25-35)_ at 10~100 μM for 48 h induced 18%~43% cellular death in a dose-dependent manner (*p* < 0.05). Aβ_(25-35)_ treatment at 25 μM induced nitrite oxide (NO) release, elevated superoxide production, and reduced the distribution of cells in the S phase. Preincubation of C13NJ with 100 μM folic acid protected against Aβ_(25-35)_-induced cell death, which coincided with a reduction in NO release by folic acid supplements. NSC47 at a level of 50 μM protected against Aβ_(25-35)_-induced cell death and reduced Aβ-promoted superoxide production (*p* < 0.05). Folic acid in combination with NSC47 at their cytoprotective doses did not synergistically ameliorate Aβ_(25-35)_-associated NO release, superoxide production, or cell cycle arrest. Taken together, folic acid or NSC treatment alone, but not the combined regimen, protected against Aβ_(25-35)_-induced cell death, which may partially, if not completely, be mediated by free radical-scavenging effects.

## 1. Introduction

Although the etiology of Alzheimer’s disease (AD) is not clearly understood, the neurodegenerative process is thought to involve extracellular plaques of β-amyloid (Aβ) and neurofibrillar tangles of tau proteins which accumulate in brain tissues [1,2]. Elevated oxidative stress is associated with the deposition of Aβ peptide, which induces neurotoxicity [3]. Various neuronal populations are differentially susceptible to oxidative damage associated with Aβ peptide challenge. Brain microglial cells, the principal immune cells in the central nervous system (CNS) which protect against microorganism invasion, are vulnerable to Aβ-induced toxicity [4]. At an early stage of Aβ-protein deposition, microglial cells are activated to release toxic immunocytokines such as nitrite oxide (NO) and products of superoxide which exert neurotoxicity [5,6]. Microglial cultures were found to clear up AD’s Aβ peptides [7]. The Aβ-induced oxidative immunotoxicity by glial activation and/or by promoting cell death was proposed to be involved in the neurodegenerative process.

Folate serves as both a carbon donor and acceptor in the *de novo* synthesis of thymidylate and amino acid interconversion, which are critical for cell proliferation and neuronal development [8]. Deprivation of folate in human neuroblastoma cells induces elevated oxidative stress, alters cytosolic calcium, and induces apoptotic death [9]. Cultivation of hippocampal cells in folate-deficient medium promotes cell death [10]. Folate supplementation, on the other hand, is associated with improving memory deficits among cognitively impaired subjects. Higher folate intake is correlated with lower risks of AD [11,12]. Folic acid (pteroylmonoglutamic acid) is the molecular form used to fortify foods and dietary supplements. It was proposed that folic acid supplements can scavenge peroxyl radicals, azide radicals, and hydroxyl radicals in an *in vitro* radical reaction model system [13]. Growing evidence suggests a potential role of folic acid in *in vivo* and *in vitro* antioxidant actions [14,15]. Those studies raised the possibility that folic acid supplementation might be effective in protecting against Aβ-induced toxicity through its antioxidant action, although a direct link has not been established.

A series of chemical compounds (NSCs) with a piperidine structure was newly synthesized according to the molecular characteristics of numerous bioactive compounds [16,17]. The bioactive functions of NSCs were first demonstrated by Chou *et al.*; NSCs enhanced cellular proliferation of peripheral blood mononuclear cells and elevated interleukin (IL)-2 and interferon (IFN)-γ production [18]. Another study showed that treatment of synapse neurons with NSCs ameliorated glutamate release [19]. Among NSCs, NSC42 has the skeleton of an indolizidine and NSC43 has the skeleton of a quinolizidine. It is well known that indolizidines and quinolizidines are important frameworks of many natural products, and have many interesting biological activities [20]. NSC47 belongs to a less-common framework of pyridoazepines, but some pyridoazepines were found to have very strong insecticidal activities [21], and were studied as potential dopamine D1 receptors [22] and nicotinic acetylcholinergic receptor ligands [23]. Synthesis methods for structures like NSC42, -43, and -47 were previously reported by our lab [24,25]. Although a few functional properties of NSC42, -43, and -47 were reported, it remains unclear if such bioactive NSCs possess any antioxidant activity to protect against Aβ-induced cytotoxicity. In light of accumulating evidence of the plausible roles of folic acid supplementation and NSCs in neuronal protection, we hypothesized that folic acid and NSCs, alone or in a combined regimen, can protect microglial cells against Aβ-induced oxidative cytotoxicity. A human microglial cell line (C13NJ) was used as the experimental model to test this hypothesis. The 11-amino acid fragment of the Aβ peptide, Aβ_(25-35)_, located in the hydrophobic domain at the C-terminal end of Aβ_(1-42)_, was shown to mimic some of the pathological processes in the AD brain [26]. Markers of cell death, elevated oxidative stress including NO release and superoxide synthesis, and cell cycle arrest were measured to explore the working mechanisms.

## 2. Results

### 2.1. Aβ_(25-35)_-Induced cytotoxicity of C13NJ cells

**Figure 1:**
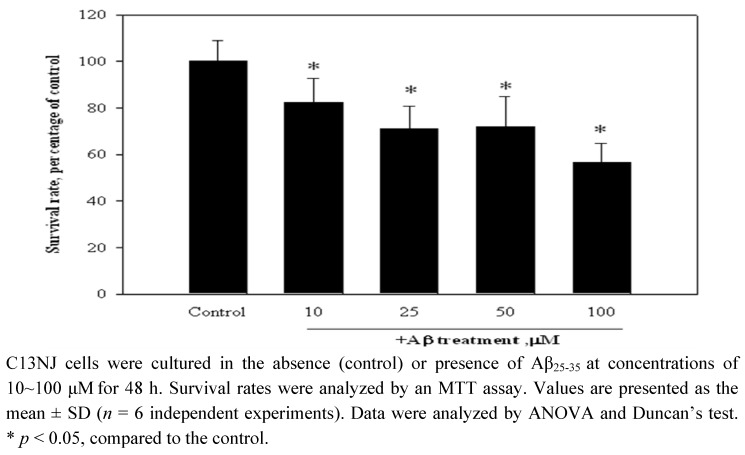
Effects of the β-amyloid (Aβ)_(25-35)_ peptide on the survival of microglial cells.

The effects of the Aβ peptide on C13NJ cell viability were studied. The data are presented in Figure 1. When treated with Aβ_(25-35)_ for 48 h, C13JN cells underwent cell death in a dose-dependent manner. Survival rates of C13JN cells were reduced by 17%, 29%, 28%, and 43% as doses of Aβ_(25-35)_ treatment increased from 10 to 100 μM. In order to use a dose of Aβ_(25-35)_ peptide to induce cytotoxicity of neuronal cells [9], Aβ_(25-35)_ treatment at a concentration of 25 μM was selected to conduct the following experiments.

### 2.2. Effects of folic acid or NSCs on Aβ_(25-35)_-induced cytotoxicity of C13NJ cells

**Figure 2:**
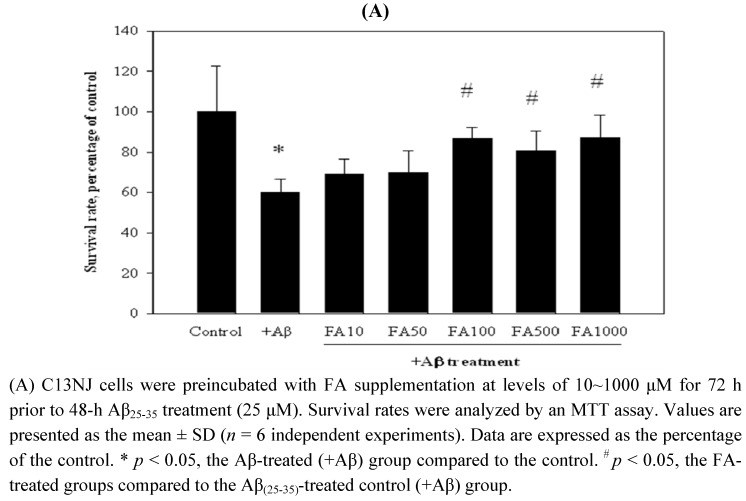

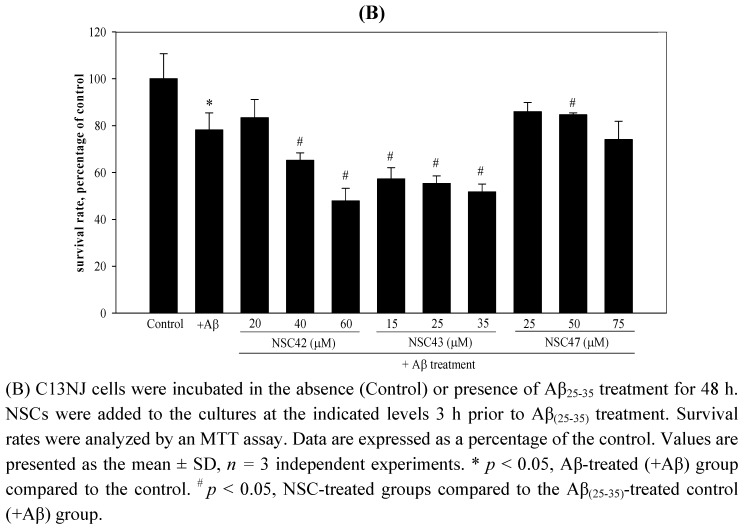
Effects of folic acid (FA) or newly synthesized bioactive compounds (NSCs) on β-amyloid (Aβ)_(25-35)_-induced cytotoxicity.

The protective effects of folic acid and NSCs against Aβ_(25-35)_-induced cytotoxicity were investigated using an MTT assay (Figure 2). Supplementation of folic acid at levels of 10~1000 μM appeared to increase the survival rate of Aβ_(25-35)_-treated microglial cells. Preincubation of C13NJ cells with folic acid at 100, 500, and 1,000 μM for 72 h significantly protected C13NJ cells against Aβ_(25-35)_-induced cytotoxicity (Figure 2A). The protective effects of NSCs (NSC42, -43, and -47) against Aβ_(25-35)_-induced cytotoxicity were studied. Test doses of each NSC compound were selected based on the anti-inflammatory actions of their analogs in human lymphocytes as previously described (13). As shown in Figure 2B, NSC47 treatment at 50 μM was effective in protecting against Aβ_(25-35)_ peptide-induced cell death. Treatment of C13NJ cells with NSC42 at 40~60 μM or with NSC43 at 15~35 μM aggravated Aβ_(25-35)_-induced cytotoxicity.

### 2.3. Effects of folic acid or/and NSC47 on NO production of Aβ_(25-35)_-treated C13NJ cells

**Figure 3:**
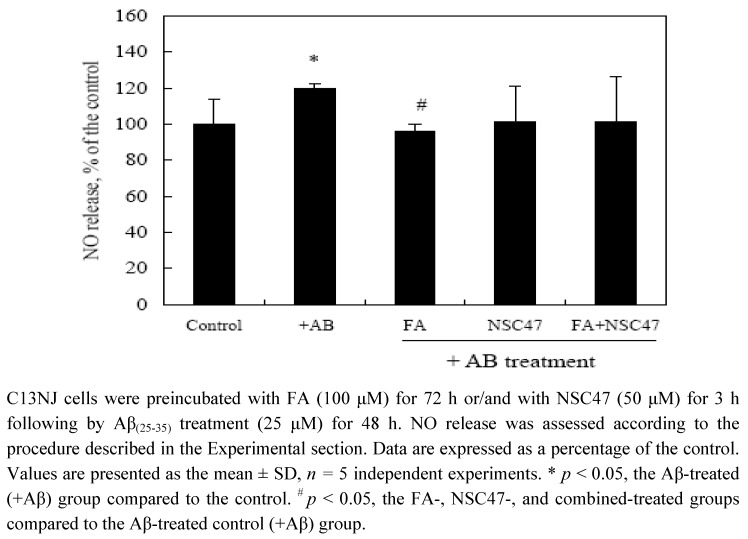
Effects of folic acid (FA) or/and NSC47 on nitric oxide (NO) production by β-amyloid (Aβ)-treated cells.

To understand if the protective effects of folic acid and NSC47 against Aβ-induced cytotoxicity are associated with oxidative modification, cellular release of nitric oxide (NO) with the various treatments was measured (Figure 3). When treated with Aβ_(25-35)_ for 48 h, C13JN cells released a significantly higher level of NO than the controls. Folic acid supplementation at a cytoprotective level (100 μM) significantly reduced NO production of Aβ-treated C13NJ cells (*p* < 0.05). NSC47 at a cytoprotective level (50 μM) reduced NO release of Aβ-treated cells, yet it did not reach statistical significance. When FA and NSC47 were combined at a cytoprotective level, synergistic effects of the combined regimen against NO release of Aβ-treated C13NJ cells were not observed. 

### 2.4. Effects of folic acid or/and NSC47 on superoxide production of Aβ_(25-35)_-treated C13NJ cells

**Figure 4:**
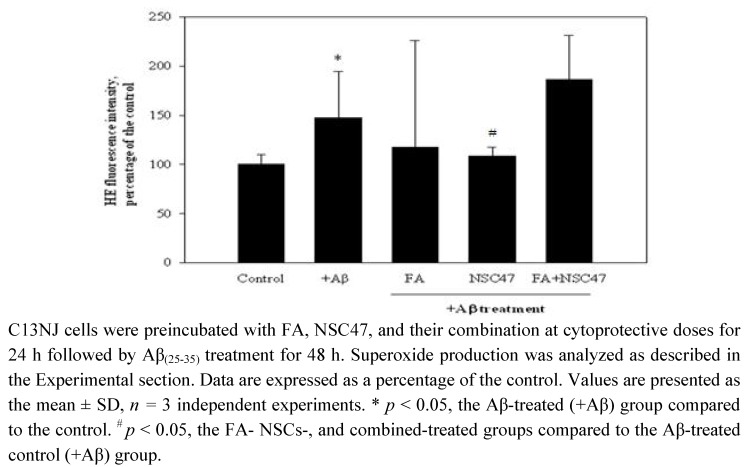
Effects of folic acid (FA) or/and NSC47 on superoxide production by β-amyloid (Aβ)-treated C13NJ cells.

Oxidative stress was assessed by measuring differential levels of superoxide production among the experimental groups (Figure 4). Previous studies revealed that preincubation of cells with folate supplements for 24~72 h or with NSC47 for 3~24 h produced similar protective effects against Aβ-induced cytotoxicity and NO release (data not shown). A time period of 24 h was selected to study the protective mechanisms of the two molecules against Aβ-induced superoxide production. Preincubation of Aβ-treated C13NJ cells with folic acid at a cytoprotective level did not affect superoxide production by Aβ-treated cells. NSC47 at a cytoprotective level significantly reduced superoxide levels of Aβ-treated cells. Combining folic acid with NSC47 did not significantly diminish superoxide levels of Aβ-treated C13NJ cells.

### 2.5. Effects of folic acid or/and NSC47 on the cell cycle of Aβ_(25-35)_-treated C13NJ cells

**Figure 5:**
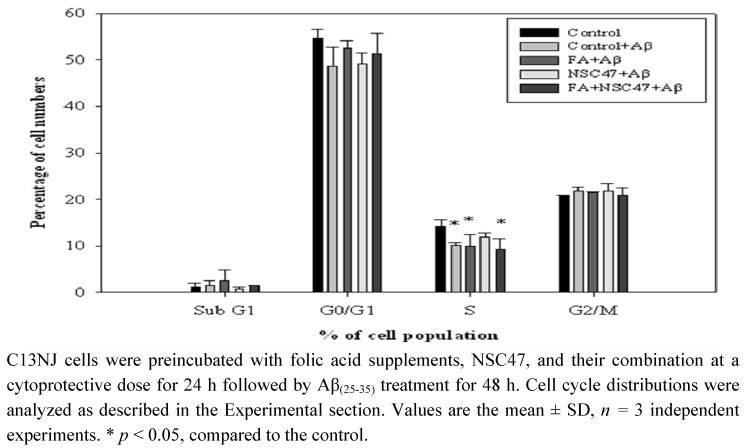
Effects of folic acid (FA) or/and NSC47 on the cell cycle distribution of β-amyloid (Aβ)-treated C13NJ cells.

As oxidative stress may signal modulation of the cell cycle of dividing cells, we further investigated the effect of folic acid or/and NSC47 on the cell cycle of Aβ-treated C13NJ cells. Figure 5 shows that Aβ challenge significantly reduced the percentages of cells in the S phase (the phase of DNA synthesis), suggesting that Aβ-induced cytotoxicity of C13NJ cells may be partially mediated by affecting DNA synthesis. Preincubation of Aβ-induced C13NJ cells with NSC47, but not with folic acid or the combined regimen, restored the proportions of S-phase cells back to the value of control cells.

## 3. Discussion

Our data revealed that treatment of human microglial C13NJ cells with the Aβ_(25-35)_ peptide promoted cell death, which was partially, if not completely, mediated by NO production, elevated superoxide generation and disruption of cell cycle progression. These findings are in agreement with results of numerous human and animal studies, which showed that synthetic Aβ peptide activation of microglia induces secretion of cytotoxic agents such as reactive oxygen intermediates [4,5,6,27]. Although microglial cells act as primary immune effector cells of the CNS which take up and degrade Aβ [28], elevation of such harmful cytotoxic substances at a low level of Aβ challenge may transform microglia to cytotoxic effector cells, which is commonly found in an inflammatory state in Alzheimer brains [29]. At a high dose of Aβ, overproduction of oxidative stress may lead to cytotoxicity to microglia, thus affecting cell cycle progression. At the lethal dose of 25 μM of Aβ_(25-35)_ treatment, the viability of microglia was significantly reduced. Consistently, that level of Aβ_(25-35)_ treatment also induced neuronal cytotoxicity [9].

We found that folic acid supplementation was effective in protecting human microglial cells against Aβ-induced cell death and NO release. Mechanisms through which folate might impact suppressed production and/or increased degradation of NO are not known. NO is synthesized by a specific enzyme, NO synthase (NOS), the activity of which increases in cerebral microvessels during AD development [30]. It was reported that folic acid treatment of SOD1^G93A^ transgenic mice suppressed activation of microglia and inhibited the expression of inducible (i)NOS in the spinal cord [31]. Alternatively, increased degradation of NO by superoxide, rather than impaired formation of NO, is predominantly observed in early atherosclerosis [32,33]. Given the fact that folic acid supplementation did not affect superoxide production of microglial cells upon Aβ challenge, the NO-superoxide interaction cycle might not contribute to the effects of folic acid in NO reduction. Determining whether the protective effect of folic acid against NO release of Aβ-treated microglia is linked to altered expression of iNOS requires further studies.

Preincubation of microglial cells with NSC47 protected against Aβ-induced cell death, superoxide generation, and disruption of cell cycle progress. The data suggested that NSC47 may act as an antioxidant to scavenge superoxides. The mechanisms by which NSC47 scavenges superoxide of Aβ-treated microglial cells are not clear. Intracellular superoxide levels can be elevated by several enzyme systems, including xanthine oxidase, uncoupled NOS, complexes I and III of the mitochondrial electron transport system, and nicotinamide adenine dinucleotide phosphate (NADPH) oxidases. NADPH oxidases are a family of protein complexes believed to be responsible for the localized and limited production of superoxide radicals [34,35]. Alternatively, Aβ-associated elevated superoxide generation may result from impaired antioxidant defense and/or superoxide generation elicited by mitochondrial dysfunction [36]. Mitochondrial dysfunction including impaired respiration and depolarization of the mitochondrial membrane potential may induce a vicious cycle of superoxide production and release of apoptotic signals which promote cellular death [37]. Determining whether NSC47 can block the activity of NADPH oxidase and/or modulate mitochondrial function to scavenge Aβ-induced superoxide warrants further studies.

We found that folic acid in combination with NSC47 at their individual cytoprotective doses was unable to act synergistically to scavenge NO or superoxide levels of Aβ-treated microglial cells. The reasons are unclear at the present time. We speculated that folic acid and NSC47, when added together to the culture medium, may interact or compete with each other for cellular uptake. In that case, the combined molecules might not achieve their individual dose effectiveness inside cells to scavenge free radicals. The fact that folic acid and NSC47 have similar bicyclic ring structures [21,24,25] suggests that interactions between these two bioactive molecules for cellular uptake may be possible. On the other hand, the current cytoprotective dose of folic acid supplement (100 μM) was not effective in intracellular superoxide scavenging, which may provide an alternative explanation of why the combined molecules did not have a synergistic effect on superoxide levels of Aβ-treated microglial cells. It was reported that suppression of superoxide production was folic acid-dependent with the most effective dose at the high level of 1,500 μM in a macrophage reference model [38]. This concentration is 15 times greater than the concentration used in the present study. A high dose of folic acid supplementation was required to resume mitochondrial respiratory function in Cu^2+^-stimulated primary hepatocytes [39]. Further studies are warranted to investigate the effective dose of folic acid alone or in combination with NSC47 to completely scavenge Aβ-induced radicals in microglial cells.

## 4. Experimental

### 4.1. Preparation of NSC, folate, and the Aβ_(25-35)_ solution

NSCs were prepared and characterized according to the method of Chou *et al.* [24,25]. The NSCs were dissolved in dimethyl sulfoxide (DMSO) to a concentration of 100 mM and then stored at 4 ºC. A stock solution of 10 mmol/L folic acid (FA: pteroylmonoglutamic acid) was prepared by dissolving 44 mg folate in 10 mL of a NaHCO_3_ (10 g/L) solution. Aβ_(25-35)_ was dissolved in 10 mg/mL double-distilled water (ddH_2_O), incubated at 37 °C for 1 week, and then stored at -20 °C.

### 4.2. Culture of human microglial cells and experimental treatments

Human microglial C13NJ cells were a generous gift from Prof. Kuo Yuh-Chi (Department of Life Science, Fu-Jen University). Cells were cultured in Dulbecco’s modified Eagle medium (DMEM) containing 10% fetal bovine serum, 10 ml/L penicillin + streptomycin, 5 ml/L fungizone, and 0.22% NaHCO_3_ at 37 °C and 5% CO_2_. Cells were maintained in a humidified 5% CO_2_ atmosphere at 37 °C. To evaluate the effective dose of Aβ_(25-35)_ peptide for inducing cellular cytotoxicity, C13NJ cells were treated with the Aβ_(25-35)_ peptide for 48 h at various concentrations of 25~100 μM. For supplemental FA and NSC experiments, C13NJ cells were preincubated with FA for 24~72 h or with NSCs for 3~24 h at designated concentrations, following Aβ treatment in the absence of FA or NSCs. Previous studies showed that preincubation of cells with folate supplement at 100 μM for 24~72 h or with NSC47 at 50 μM for 3~24 h produced similar protective effects against Aβ-induced cytotoxicity and NO release (data not shown). A time period of 24 h was selected to study the protective mechanisms of the two molecules against Aβ-induced superoxide production and cell cycle arrestment.

### 4.3. MTT test

C13NJ cells were incubated at 1.2 × 10^4^ cells/well in a 96-well culture plate for 6 h, then treated with various concentration of Aβ_(25-35)_ for 48 h. After washing with phosphate-buffered saline (PBS), 200 μL 0.5 mg/mL MTT was added, then incubated at 37 °C for 3 h. The supernatants were removed, then mixed with 200 μL/well DMSO for 15 min. The plate was read on an enzyme-linked immunosorbent assay (ELISA) reader at 570 nm.

### 4.4. NO release assay

C13NJ cells (5 × 10^5^ cells) were cultured with FA or/and NSC at designated concentrations and time periods, then treated with 25 μM Aβ_25-35_ for 48 h. Cultured medium at 80 μL was mixed with 200 μL/well Griess reagent (1% sulfanilamide and 0.1% N-1-naphthylethylene diamide dihydrochloride and 2.5% H_3_PO_4_) for 5 min. Nitrite concentrations were determined at 540 nm on an ELISA reader, and then calculated with a standard curve of sodium nitrite.

### 4.5. Intracellular reactive oxygen species (ROS) levels

Intracellular ROS, particular superoxide production, were assayed using the fluorescent dye, hydroethidine (HE) [40]. HE oxidation is particularly sensitive to superoxide anions. Thirty minutes before Aβ treatment was terminated, HE (5 mmol/L) was added to cultured cells. At each indicated time point, cells were harvested, and the fluorescence intensity of intracellular hydroethidine (with emission at 585 nm) was monitored on a Coulter EPICS XL-MCL flow cytometer (Miami, FL, USA).

### 4.6. Cell cycle analysis

After 48 h of Aβ treatment, control cells and cells in each group were harvested, fixed in ice-cold 100% ethanol, and treated with ribonuclease A (500 mg/L) and 0.5% Triton at 37 °C for 60 min. Cells were then stained with propidium iodide (PI; 50 mg/L for DNA staining) for 20 min. After centrifugation, the pellet was resuspended in PBS with the same concentration of PI for 10 min. Cellular DNA in 10,000 cells was analyzed in a Coulter EPICS XL-MCL flow cytometer. The percentage of cells in each phase of the cell cycle was determined by the WinMDI 2.8 program.

### 4.7. Statistical analysis

All data are presented as the mean ± SD. One-way analysis of variance (ANOVA) and Duncan’s test (SPSS software) or Student’s *t*-test were used for comparisons among groups. A significant difference was indicated when the *p* value was < 0.05.

## 5. Conclusions

In summary, treatment with folic acid or NSC47 alone protected against Aβ_(25-35)_-induced cell death and elevated oxidative stress in human microglial cells. The present study provides new mechanistic insights on the roles of folic acid and NSC47 in the protection against Aβ_(25-35)_-induced microglial toxicity through antioxidant activities. Folic acid in combination with NAC47 at their respective cytoprotective doses did not have a synergistic protective effect on Aβ-induced oxidative stress, which warrants further studies.
